# When nature frowns: A comprehensive impact assessment of the 2012 Babessi floods on people’s livelihoods in rural Cameroon

**DOI:** 10.4102/jamba.v7i1.197

**Published:** 2015-11-27

**Authors:** Roland A. Balgah, Gertrud Buchenrieder, Innocent N. Mbue

**Affiliations:** 1 Department of Agribusiness Technology, University of Bamenda, Cameroon; 2 Department of Agriculture and Rural Development, Bamenda University of Science and Technology, Cameroon; 3 Institute of Agricultural and Nutrition Sciences, Martin-Luther-University Halle-Wittenberg, Germany; 4 Department of Occupational Hygiene, Safety and Environment, University of Douala, Cameroon

## Abstract

Floods are the most common natural disasters worldwide. Much of the growing literature on the impact of floods, especially in developed countries, and to a lesser extent in rural areas of developing countries, concentrates on economic rather than a comprehensive assessment of combined effects on people’s livelihoods. Holistic floods impact assessments are often done long after the shock, raising problems of data reliability following long recall periods, although post-disaster needs assessments when carried out earlier can facilitate appropriate disaster recovery, relief and reconstruction activities. We applied the sustainable livelihoods framework as a comprehensive approach to assess the impacts of the Babessi floods in 2012 on livelihoods in rural (north western region) of Cameroon 6 weeks after the floods. Using a structured questionnaire, data was collected from victims before and after the floods, using recall methods. A matched sample of nonvictims randomly selected from the same village as the victims was used to assess vulnerability to the floods by household type. Floods were found to have serious economic, social, human and food security impacts on victims. Both government and nongovernmental support were jointly crucial for household recovery. Comparatively observed high levels of recovery were attributed to the low loss of human lives. The article concludes with the need for comprehensive approaches to floods impact assessments. The need for combining formal and informal instruments in post-disaster management in rural areas is also emphasised.

## Introduction

Increasing frequency and severity of natural disasters affect livelihoods especially of the poor around the world, further creating poverty traps (Carter & Barrett 2006). About 240 million people’s livelihoods are estimated to have been disrupted by natural disasters worldwide between 2000 and 2005 (Feron 2012). More people were killed by natural disasters in 2013 (21 610) compared to 2012 (9710) (Guha-Sapir, Hoyois & Below 2014). Recent estimates place annual economic losses from natural disasters between ․250 million and ․300 million (United Nations Office for Disaster Risk Reduction [UNISDR] 2015).

Floods are amongst the most disastrous form of nature’s sudden, usually unpleasant reaction, impacting the human race worldwide. In fact, floods are the most common natural disasters in Europe and Africa as a consequence of, but not necessarily limited to, climate change (Guha-Sapir *et al.* 2011, Guha-Sapir *et al.* 2014, World Health Organization [WHO] 2002). In the last three decades, over 2000 floods recorded worldwide affected and distorted the livelihoods of victims. In total, 56.1% of all disasters recorded by the Center for the Epidemiology of Disasters (CRED) in 2010 were of floods origin, affecting 189 million people (Guha-Sapir, Hargitt & Hoyois 2004, Guha-Sapir *et al.* 2011, Guha-Sapir *et al.* 2014). Floods are estimated to have increased worldwide by 145.1 percentage points in 2010 compared to the annual averages between 2000 and 2009 (Guha-Sapir *et al.* 2011). Resulting losses were reported mainly in the agricultural production sector. Such damages have negative effects on the livelihoods of victims, especially in developing countries where a majority of households still depend on smallholder agriculture for survival (Balgah & Buchenrieder 2011, Barrett, Sherlund & Adesina 2008).

In recent times, economic growth and social stability in Africa have witnessed increasing threats from unprecedented upsurge of floods. In 2010 for instance, floods alone accounted for 82.6% of all disasters on the African continent, up from 66.5% in 2009. This accounted for a total economic loss of ․59.2 million (Guha-Sapir *et al.* 2011). The rapid increase in flood frequency has been attributed to climate change effects such as El Ninos, increasing sea levels (estimated at 10 cm – 20 cm in the 20th century), sudden heavy downpours (Nicholls 2002), and uncontrolled urbanisation and deforestation (Abuaku *et al.* 2009, Nirupama & Simonovic 2007). Reliable estimates suggest that more floods will be witnessed on the globe in the future. Rural areas especially in Africa have been identified to be particularly vulnerable to increased incidence of flooding, because protecting them is often deemed less economically and politically plausible relative to urban areas (Posthumus *et al.* 2009, Wilby, Beven & Reynards 2008).

The need for increasing research on disasters in general and on flood dynamics in particular, especially in rural areas of developing countries, has therefore been recognised (Okuyama & Santos 2014). This has been demonstrated by the rapid increase in flood-related analysis worldwide. Whilst floods impact assessments in rural areas are on the increase, they mostly focus on the economic component of livelihoods. Their impacts on private property have been frequently acknowledged (e.g. Francisco [2014] for Manila in the Philippines). This article contributes to the flood literature, by comprehensively assessing the economic, human, social and food security effects on victims, using an empirical case study from rural Cameroon.

This article will proceed as follows. The next section will present a succinct review of the literature. Emphasis is placed on the impacts of floods, and on the applicability of the livelihoods framework for floods analysis. The materials and methods applied in the research are then presented. This is followed by a presentation of the empirical results. Discussions on the case study and the implications for research and policy will conclude the article.

## State of the art of the floods impact literature

Increasing global frequency of floods in the last three decades creates growing concern. As mentioned already, in 2010 alone, floods were estimated to have increased by 145.1 percentage points compared to the annual averages between 2000 and 2009. In the same year, 56.1% of all disasters recorded globally were of floods origin. These floods caused damages to over 2.6 billion people globally, resulting in direct economic losses of ․46.9 billion (Guha-Sapir *et al.* 2004; Guha-Sapir *et al.* 2011; International Strategy for Disaster Reduction [ISDR] 2010) The agricultural production sector particularly in developing countries was affected most significantly (Balgah & Buchenrieder 2011; Barrett *et al.* 2008). Considering that the African economy still strongly depends on agriculture – and that the relative share of the poor and hungry is highest in this continent, the need to understand the dynamics of floods, their impacts and management strategies on the continent is urgent.

In the disaster management literature, it is generally agreed that the impacts of extreme events, such as floods, are contingent on their frequency and the degree of correlation amongst affected individuals (covariate or idiosyncratic) or with other risks (bunched), the exposure of the livelihood system and the risk management strategies applied by victims (Holzmann, Sherburne-Benz & Telsuic 2003; Karin, Sudharshan & Siegel 2001; Posthumus *et al.* 2009). Significant efforts have been made by research to understand and explain how the frequency, correlation, exposure, and management strategies influence the impact of extreme events (Abuaku *et al.* 2009; Balgah & Buchenrieder 2014; Holzmann *et al.* 2003; Karin *et al.* 2001).

Assessing impacts on natural disasters such as floods have often concentrated on the economic aspects and the determinants of economic losses (Abuaku *et al.* 2009; Benson & Clay 2004; Cuaresma 2009; Posthumus *et al.* 2009). Abuaku *et al.* (2009), for instance, report a ․30 billion in economic damages following the July 1998 floods in China, across 29 provinces. The agricultural sector was highest hit, with 21 million hectares of land flooded. Posthumus *et al.* (2009) conclude in their study of the 2007 summer floods in England that over 80% of agricultural fields were flooded, leading to losses in crop yields, product quality, increased farm inputs and harvesting costs, estimated at an average financial loss of UK£ 8915 per farm. Without emphasising a comparative analysis, both case studies portray the agricultural sector as highly vulnerable and most exposed economic sector, when floods occur.

Apart from direct impacts, for instance, on agricultural production and assets, floods are known to have serious direct and indirect social, psychological and other effects on victims. Such effects may be caused by the loss of human lives^1^, the spread of faecal and vector-borne diseases, mental disorders and other forms of psychosocial traumas that often accompany floods or persist after such events occur (Abuaku *et al.* 2009; Neira *et al.* 2008). This calls for impact assessments that go beyond the economic aspects. Abuaku *et al.* (2009) for instance report a higher incidence of diseases amongst persons aged 45 and above after the 1998 floods in Hunan, China. Molua (2009) and Posthumus *et al.* (2009) observe some negative perceptions of flood victims to government intervention strategies in Cameroon and England respectively. Negative perceptions can have serious effects on short, medium and long term efforts to deal with floods. In summary, the challenges expected to accompany climate change in general and increasing floods in particular seem gloomy for the agricultural sector (Intergovernmental Panel on Climate Change [IPCC] 2014). Whilst it is true that post-disaster needs assessments (PDNA) can support disaster recovery, relief and reconstruction activities immediately after floods occur (Guha-Sapir & Lechat 1986; Ingram *et al.* 2006; Malilay, Flanders & Brogan 1996), comprehensive analytical approaches can potentially provide stronger plausibility to floods impact assessments, better adaptability to climate variability and adequate policy options for flood risk management. Increasingly, impact assessments benefit from spatial analysis to enhance completeness (e.g. Appeaning Addo & Adeyemi [2013] for application on a coastal community in Accra Ghana, or Lawal & Arokoyu [2015] for south western Nigeria). This goes beyond the current research. We limit our research in understanding to what extent livelihoods approach can be applied to capture floods impacts amongst victimised households, based on a case study of flood victims in rural Cameroon.

## Linking floods impact assessments to the livelihoods framework

The sustainable livelihoods framework has been widely used to analyse how households combine different livelihood resources and strategies, under specific (institutional, structural and vulnerability) contexts, to achieve certain livelihood outcomes (Department for International Development [DFID] 1999; Kydd 2002; Scoones 1998). In any given (vulnerability) context, the framework allows for an analysis of how different livelihood resources (natural, human, social, physical and financial capitals) can or are combined in some livelihood strategies, to attain certain outcomes (Scoones 1998). When floods occur for instance, it is the availability of the different forms of capitals and the ability of the victims to combine them (or not) based on the existence and functioning of (formal and informal) institutions, that will determine the level of the impacts (Holzmann *et al.* 2003). Thus, whilst this framework has generally been used to understand and tackle poverty (Kydd 2002), it is potentially applicable in assessing the impacts of natural disasters, such as floods. Impact assessments based on the livelihoods framework often pay attention to the evolution or strategic changes in livelihood assets – natural, physical, financial, human and social capital assets (Scoones 1998). Floods often impact these assets amongst victims, and have the capacity of rendering stable livelihoods unsustainable. The connection between the occurrence of floods and livelihood sustainability seems quite obvious. The livelihoods framework can therefore by applied in modelling the impacts of floods, especially on livelihood assets. In its entirety, the livelihoods framework consists of the vulnerability context (i.e. shocks, trends and seasonal changes), livelihood assets, institutions and organisations (alternatively called transforming structures and processes), livelihood strategies and livelihood outcomes (DFID 1999; Scoones 1998). A complete livelihood analysis will therefore look at the interactions between these different components.

Many attempts have been made to apply the entire livelihoods framework or part of it thereof, to assess the impacts of project treatments on livelihood outcomes (Cuaresma 2009; Kydd 2002; Meinzen-Dick & Adato 2001). Meinzen-Dick and Adato (2001), for instance, applied the entire framework to assess the impact of agricultural research on poverty and the implications for integrated natural resource management in IFPRI (International Food Policy Research Institute) projects in Bangladesh, Kenya and Zimbabwe. They concluded that agricultural research had a significant impact on livelihood outcomes. The livelihoods framework was seen in the case study as a tool that resonates well with researchers who have experience in rural life and a good understanding of poor people’s experiences. Lesser efforts have been made to apply this framework in modelling natural disasters.

This article applies the framework to assess the impact of the 2012 Babessi floods on livelihoods in rural Cameroon, by examining differences in livelihoods assets, before and after the floods. A special interest is also placed on the role of endogenous mechanisms (or institutions) in managing floods on livelihood outcomes. This rural case study intends to contribute to the discourse on the effects of natural disasters in general and floods in particular on livelihoods in sub-Saharan Africa where more than 30% of the population are absolutely poor, and are likely to be pushed into a vicious poverty cycle by increasing frequency of floods (Naude 2010).

## Materials and methods

### Problem background and setting

On 09 September 2012, Babessi, one of the 13 villages of Ngoketunjia Division in the north west region of Cameroon experienced a serious shock, when it was suddenly struck by an ugly, 30 min floods (18:30–19:00) that destroyed 56 houses and rendered 26 families completely homeless in the three most affected residential areas of Chui, Touncho and Mbaw (Loh 2012). Whilst the duration of the floods was relatively short and human losses numerically low, initial losses in agriculture were estimated to be many tens of millions of Franc CFA (1 US․ is approximately 450 FCFA) (Adamu 2012). The cause of the floods has been attributed to unusual heavy rains in the hillside of neighbouring Jakiri subdivision and poor drainage in Babessi itself (Loh 2012). Most of the affected households took refuge with family members and friends – initially depending on these informal mechanisms to manage the covariate shock (Balgah & Buchenrieder 2014). Appreciable amounts of humanitarian, physical, financial and psychosocial support was provided to victims by both state (formal) and nonstate (informal) institutions such as elite groups and nonprofit organisations (Adamu 2012; Loh 2012). At the time of this research (about 6 weeks after the floods) a local disaster management commission existed, which was made up of both victims and nonvictims alike and coordinated by the Divisional Officer for Babessi subdivision, who is a direct representative of the Head of State. This local disaster management institution moderated the coping process for victims.

### Methodology

The aim of this research was to assess the impact of the floods on the livelihoods of victims in Babessi Subdivision in the Northwest Region of Cameroon, using the livelihoods framework. A structured questionnaire was applied to collect data from both victims and nonvictims; 38 of the 56 victimised households participated in the survey. The remaining 18 households simply did not want to participate in the survey. Nevertheless, the research attained an almost 70% coverage of all victims. Moreover 24 purposively selected nonvictimised households in the same village also participated in the survey as a control group. At the time of the interview, many households that had been categorised by the above-mentioned local disaster management commission as nonvictims, identified themselves to the survey team as victims because they hoped to benefit from any humanitarian support coming to the village and destined for the victims. Clearly, these households could then not be included in the control group. Thus, the sample for this research was limited to those who truly, self-identified themselves as nonvictims. This accounts for the rather small sample size of nonvictims with only 24 observations. The structured questionnaire allowed the team to capture the livelihood situation of victimised and nonvictimised households before and after the floods. The research made use of the asset portfolios of the sustainable livelihoods frameworks for the comprehensive impact assessment (DFID 1999; Scoones 1998). Economic impacts were elicited as a combination of physical assets (i.e. selected household and livestock assets) and financial assets (household cash held at household at the time of the flood) lost to the disaster. Natural assets were proxied by the ability of the household to provide own food from own natural food stocks. Social impacts were captured by assessing the psychosocial suffering resulting from the loss of human lives; valued books destroyed, and household perceptions on the level of recovery and willingness to relocate to safer areas on the basis that there are social mechanisms to support relocation. Human assets were assessed through household size (eventual labour loss is implicit) and age of household head.

Field research took place 6 weeks after the disaster, from 29 to 31 October 2012. Data collection was done at the household level, although this was mainly done through the household head. Qualitative methods such as observations, key informant interviews and participatory discussions were applied to complement the questionnaire. Data was analysed using SPSS (The Statistical Package for Social Sciences) and Excel.

## Results

### Socioeconomic characteristics of sampled households

The descriptive statistics on selected socioeconomic characteristics of sampled households are presented in Table 1. Both victimised and nonvictimised households had a similar mean household size of approximately eight members. Whilst this is not different amongst the household types, it is quite high compared to a mean household size of 5, found by previous research in other parts of the northwest region of Cameroon (e.g. Balgah & Buchenrieder 2011). This is probably due to the reported high agricultural fertility of the valley swept by the floods, which might have encouraged larger household sizes, for labour supply on family farms, as well as for the fact that the households are largely food self-sufficient. It was also revealed through participatory discussions and observations that polygamy was common in the research area. This implicitly might have contributed to the larger household sizes. On average, victimised household heads were 4 years older than the matching (nonvictimised) ones. This difference was however not significant at the 5% level. Victims on average had resided in the Babessi community 7 years more than the matching sample (26 and 19 years respectively). Since the valleys are often very fertile, it is likely that earlier residence acquired land in these areas, which have turned out to be highly exposed to floods. The annual per capita expenditures on clothing and foot wear narrows down to FCFA 25, 450 (․54) and 23, 450 (․50) for victimised and nonvictimised households respectively. According to previous research (e.g. Minten & Zeller 2000), this expenditure represents around 5% – 10% of the household expenses and increases with household income. Thus flood victims are likely to have higher expenditures (and income) than nonvictims, even if this difference is not statistically significant. This suggests that the victims are likely to have been better off than nonvictims before the floods. In other words, it is not the very poor who were affected by the Babessi floods. Although we cannot attribute it to a specific characteristic of the household, on average, flood victims reported one more idiosyncratic shock than nonvictims. However, nonvictims reported one more covariate shock on average compared to flood victims. That the households generally reported additional shocks suggests that the Babessi community might have been exposed to a number of shocks, the most important in terms of damage of which was the 2012 floods. If this conjecture holds, then both the poor and the less poor are exposed to the risk of witnessing the effects of nature’s frown in the research area. Past shock experiences are likely to have further exposed households to the floods.

**TABLE 1:** Comparative socioeconomic analysis of floods – affected and nonaffected households.

Variable	Household type	Mean	Standard deviation	*P*-value
Household size	Victimised	7.82	5.129	0.978
	Nonvictimised	7.87	7.552	
Age of household head (years)	Victimised	42.64	13.655	0.229
	Nonvictimised	38.45	12.428	
Years of residence in the community	Victimised	26.33	21.455	0.176
	Nonvictimised	19.09	19.033	
Household expenses on clothing and footwear in the last 12 months (Franc de la Communauté Financière d’Afrique)	Victimised	203 605	158 365	0.172
	Nonvictimised	187 590	158 060	
Total number of annual idiosyncratic shocks	Victimised	5.49	8.76	0.238
	Nonvictimised	3.57	3.45	
Total number of annual covariate shocks	Victimised	0.72	1.146	0.037
	Nonvictimised	1.48	1.442	

Note: All monetary amounts have been rounded to the nearest *Franc de la communauté financière d’Afrique* (FCA); 1 US ․ is equivalent to 450 FCA.

### Economic impacts of the 2012 Babessi floods

The economic impacts of the 2012 Babessi floods were assessed based on an analysis of livestock assets, selected household assets and cash holdings at the household 1 day before and after the disaster. This analysis was restricted to flood victims only. Table 2 presents the results of livestock assets. Losses significant at 5% level were reported for small ruminants (sheep, goats), pigs and poultry. Poultry recorded the highest percentage loss of 83%, followed by small ruminants and pigs (60% and 58% respectively) and cattle. That the smallholder farmer often holds a reasonable portion of his or her assets in the form of livestock (Wollmer 1997) suggests that endogenous risk management mechanisms were greatly handicapped by the Babessi floods rendering self-recovery or coping very difficult. This was further compounded by the loss of cash held at the household at the time of the floods. On average, households lost 99.8% of the cash held at home to the floods, from a mean of FCFA 53 950 before the floods to a meagre FCFA 105 after the floods. As reported by victims, survival would have been almost impossible in the first days following the floods, were it not for the intervention of friends and family members (cf. Loh 2012). This supports earlier conjectures that socially embedded, informal response mechanisms can play a crucial role in the recovery of victims, in the presence or absence of functioning state and market instruments (e.g. Balgah & Buchenrieder 2010; Bang 2013; Campbell 1999). The losses on selected household assets (Table 3) were also quite substantial. The intervention of government and nongovernmental organisations observed during the field research was therefore necessary to enhance short term coping, midterm adaptation and long term recovery processes of victimised households. This external support together with community based disaster management mechanisms can support victims to quickly recover from the economic and social impacts of the Babessi floods.

**TABLE 2:** The impact of the Babessi floods on Livestock assets.

Variable	Time frame	Mean FCFA	Standard deviation	*P*-value
Value of cattle (FCFA)	Before floods	123 075	401 615	0.099
	After floods	61 540	227 535	
Value of small ruminants (FCFA)	Before floods	144 105	401 615	0.030
	After floods	58 590	161 955	
Value of pigs (FCFA)	Before floods	135 895	246 055	0.026
	After floods	57 690	155 030	
Value of poultry (FCFA)	Before floods	51 925	67 985	0.001
	After floods	8925	16 250	
Value of other livestock (FCFA)	Before floods	3140	9175	0.092
	After floods	470	1690	

Note: All monetary amounts have been rounded to the nearest *Franc de la communauté financière d’Afrique* (FCA); 1 US ․ is equivalent to 450 FCA.

**TABLE 3:** Changes in value of selected physical assets resulting from the 2012 Babessi floods.

Variable	Time frame	Mean	Standard deviation	*P*-value
Value of television set(s)	Before floods	39 080	48 140	0.007
	After floods	7895	16 990	
Value of radio sets	Before floods	3140	9175	0.092
	After floods	465	1690	
Value of chairs	Before floods	87 270	104 675	0.022
	After floods	42 085	42 085	
Value of cupboards	Before floods	18 890	48 745	0.183
	After floods	2430	10 905	

Note: All monetary amounts have been rounded to the nearest *Franc de la communauté financière d’Afrique* (FCA); 1 US ․ is equivalent to 450 FCA.

### Impact of floods on short term food security

Short-term food security impacts were captured by assessing the average number of meals per day amongst the victims, before and after the event. It is assumed here that households who cultivate more land would be able to draw on food stocks to overcome the short term pitfalls in food security, before the eventual arrival of external aid. As shown in Table 4, the flood victims lost one meal on average after the disaster, eating twice a day, instead of three times as before the event. Whilst the before–after difference is statistically significant (*p* = 0.000), there seems to be a strong contribution to consumption smoothing by drawing on food stocks that were amply available at household level before the shock. Participatory discussions revealed that most of the maize in the barns was not swept away by the floods, and households relied on such stocks for food supply immediately after the Babessi floods. The arrival of humanitarian aid from formal and informal stakeholders soon after the floods, however quickly, regularised the food supply problem in a very short time (Adamu 2012; Loh 2012). The fairly accessible nature of the village located in the Ndop plains of Northwestern Cameroon can also be seen as a natural asset that favoured quick supply of humanitarian assistance.

**TABLE 4:** Impact of floods on food security.

Variable	Time frame	Mean	Standard deviation	*P*–Value
Number of meals per day	Before floods	2.74	0.511	0.000
	After floods	1.79	0.687	

### The social impacts of the 2012 Babessi floods

**FIGURE 1:**
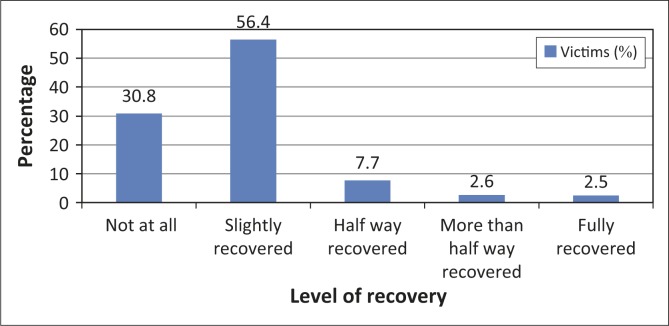
Level of recovery 7 weeks after the Babessi floods.

It is worth noting that the floods completely destroyed 26 homes and rendered over 50 families homeless (Loh 2012). Nevertheless to further examine the social impacts of the disaster, an effort was made to assess the value of loss in terms of socially important variables such as human lives and books. As presented in Table 5, substantial losses were recorded in terms of reading material destroyed by the floods. In general, even though the dead toll was statistically significant, it was lower than has been reported recently for the 2013 floods in the Philippines (Mogato 2013), or for instance for the 1986 lake Nyos gas disaster in the same region of Cameroon (Balgah & Buchenrieder 2014). The value of books destroyed by the floods was also remarkable. Measures of post-traumatic stress disorder (PTSD), that pay attention to psychosocial and psychiatric problems could be useful in further illuminating the short and long term social impacts of the disaster (Cabtree 2012; Neira *et al.* 2008). However, this was not considered within the scope of this research. Nevertheless, for a better understanding of the social impacts, the current level of recovery of victims was assessed, as well as their willingness to relocate into safer areas allocated by the Cameroon government. Figure 1 presents the results. Less than 3% of all households agree to have fully recovered 7 weeks after the floods. The majority (over 87%) were still heavily living in shock, whilst about 10% were in the process of recovery. Assuming a linear 3% recovery every 7 weeks, it will take about 5 years for all victims to fully recover. A recent study of 1986 lake Nyos disaster victims in North West Cameroon found out that only 20% of households had fully recovered after a quarter of a century (Balgah & Buchenrieder 2014). The increased awareness and preparedness of government institutions in Cameroon to respond to extreme natural events (Bang 2013) and the accessible nature of the flooded region seem to have contributed to the rapid recovery rate, as compared to victims of the 1986 lake Nyos disaster. It is also likely that victims find it much more difficult to recover from disasters with high human losses, as was the case with the 1986 Lake Nyos disaster. Also the rapid reactions of state and nonstate actors immediately after the Babessi floods and the formation of a community based flood management committee (Adamu 2012; Loh 2012) suggest that collaborative disaster management can significantly reduce the time of recovery and the social impact of disasters. According to Neira *et al.* (2008), background factors, level of exposure, social support factors and personality traits influence not only the level of PTSD amongst the disaster victims, but also the rate of recovery. This however needs to be confirmed through further research.

**TABLE 5:** Value of books and number of lives lost by households to the Babessi floods.

Variable	Time frame	Mean	Standard deviation	*P*-value
Value of books (FCFA)	Before floods	7010	34 585	0.195
	After floods	435	2060	
Number of household members	Before floods	7.3226	4.14236	0.000
	After floods	7.0938	4.27566	

Note: All monetary amounts have been rounded to the nearest *Franc de la communauté financière d’Afrique* (FCA); 1 US ․ is equivalent to 450 FCA.

**FIGURE 2:**
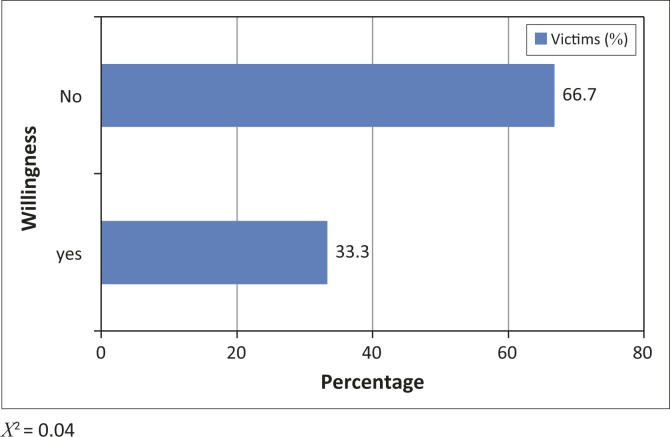
Household willingness to self-relocate out of the current disaster zone.

**FIGURE 3:**
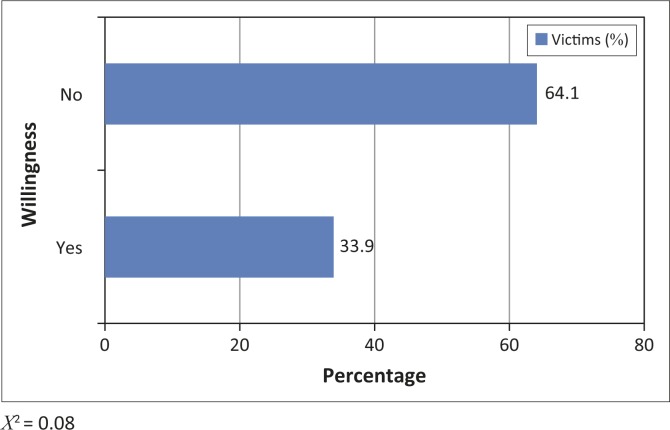
Household willingness to relocate under the influence of the national government.

An interesting aspect of the social impacts of a disaster is the willingness of victims to relocate under different conditions. The opinions of victims were sought on their willingness to self-relocate, or to relocate under the influence of the national government of Cameroon. An overwhelming majority was reluctant either to self-relocate (66.7%), or to do so as part of a government policy (64.1%). Previous research on disaster management in Cameroon suggests that such reluctance is largely due to the lack of trust in government institutions in the management of natural disasters (Bang 2008, 2013; Ngwa 1992). On the other hand, it could also be influenced by factors such as land scarcity and social attachment to the community or to engagements in the moral community. Key informant interviews revealed that dead family members are often buried inside the house in Babessi. Relocating will mean departing from loved ones. Many victims do not seem prepared to be socially detached from the dead relatives through relocation (Figures 2 and 3).

## Discussion and conclusion

Changes in climatic factors such as rising sea levels and unusually heavy down pours have made floods to become a major natural shock to reckon with, as it is mostly accompanied by devastating short and long term effects on the livelihoods of victims. Although floods impact mostly developing countries, studies on the impact of these floods on livelihoods are not commensurate with the magnitude of the problem. In addition, the long recall periods on which such studies are often based reduce the quality of the data, subsequent results and policy interventions. Case studies that go beyond economic aspects to focus on a wider comprehensive assessment of flood impacts on livelihood outcomes especially in developing countries are scarce. This article focused on bridging this gap, by assessing the impact of the September 2012 Babessi floods on livelihoods in rural Cameroon, whilst paying attention the role of formal and informal instruments in post-disaster management in a developing country context. Leaning on the livelihoods framework, impact assessment was done by mainly comparing victim situation before and after the event, using economic or financial, natural (food security), human and social indicators. A comparison of victims and nonvictims before the floods allowed for testing in parallel, the widely celebrated hypothesis that the poor are highly exposed to natural disasters. Our analyses of the results lead to a number of conclusions.

Firstly, victims were on average not poorer than nonvictims before the floods, suggesting the poorer – more exposed paradigm cannot be treated as a hard and permanent rule in every disaster situation. For flood victims in particular, it seems that everyone, poor or not is likely to be exposed, as it is often sudden.

Secondly, the 30 min Babessi floods impacted enormous economic losses on victims especially on their small livestock. That the livestock is often a reserve capital for farmers in developing countries indicates to what extent the frown of nature demonstrated through floods can punctuate existing livelihoods, and further create poverty traps (Carter & Barrett 2006; Wollmer 1997). Significant losses were also observed on the cash held by the household at the time of the floods. Household food intake dropped by one meal per day on average after the floods. Losses of human lives were reported from Babessi floods as wells as destruction of books, which can contribute immensely to human capital formation for many households. Destruction on houses was still very visible at the time of the survey. Less than 5% of the victims had fully recovered at the time of interview, nearly 2 months after disaster. This rate however shows a marked improvement as compared to other disasters studied in the region, with high losses in human lives. Major contributing factors to an acceptable level of recovery were likely the increased trust in the government obtained by the creation of a disaster management committee that involved the victims and low losses in human lives. It is very likely that trust in government institutions can be improved through collaborative disaster management mechanisms, as exemplified by the joint disaster management committee observed in the case study. This is a window of opportunity for the establishment of appropriate institutions for preventing, mitigating and coping with current floods and enhancing resilience to future ones in the region. As stated by Ahrens and Rudolph (2006) and Bang (2008, 2013), such participatory governance enhances the implementation of public policies for disaster risk reduction, enhancing the potential of national governments to promote social development, reduce susceptibility to disasters and enhance sustainable livelihoods. This view is strongly supported by Molua (2009) in his empirical study of responses to climate change amongst households in the coastal regions of Cameroon.

Thirdly, the social attachment of many victims to their current dwelling places was so strong that a large majority was not willing to self-relocate or relocate as part of a government-led programme. Issues on post-traumatic stress disorder that are crucial in assessing social impacts were not part of the study. Further research in this direction will be useful in deepening the understanding of the social impacts of the Babessi floods.

Crucial to managing floods and other forms of natural disasters is risk mapping. Participatory risk mapping can encourage both preventive and mitigative measures in flood risk zones in the country, by creating awareness and implementing timely measures. Involving the local population throughout the process will make the expected difference. As mentioned by TeLinde *et al.* (2011), moving away from flood defense and coping towards preventive and mitigative risk management approaches is more appropriate, as increasing floods is an inevitable consequence of climate change. For this to happen, increased focus has to be placed on spatial planning, preflood risk management measures and estimates, and developing appropriate institutional framework. These processes will enhance the sustainability of livelihoods in many floods – prone areas in developed and developing countries. For Cameroon that is already witnessing an increase in floods, the time to act is now.

The sample size for nonvictims was relatively small, as most of them took advantage of the situation to declare themselves as victims in an attempt to capture any benefits targeted towards victims. Further research in the region should therefore seek to increase the sample size of this group in order to enhance the quality of the comparative analysis. An impact assessment according to gender could also reveal the differentiated impacts for different household members. An emphasis on PTSD in future research can further bring to light the psychosocial impacts of floods amongst the victims.

## Footnotes

1 We refrain in this article from giving a monetary value to human lives, although labor is a production factor and thus a disease burden or loss of it is negatively affecting the economic livelihood. For completeness, it may be mentioned that a measure to give overall disease burden or early death a value is given with the common metric, the so-called disability-adjusted life year (DALY).
